# Attomole-per Cell Atomic Mass Spectrometry Measurement of Platinum and Gold Drugs in Cultured Lung Cancer Cells

**DOI:** 10.3390/molecules26247627

**Published:** 2021-12-16

**Authors:** Wioletta Jakubczak, Maja Haczyk-Więcek, Katarzyna Pawlak

**Affiliations:** Faculty of Chemistry, Warsaw University of Technology, Noakowskiego 3, 00-664 Warsaw, Poland; wiolettajakubczak@gmail.com (W.J.); maja.haczyk@gmail.com (M.H.-W.)

**Keywords:** cytotoxicity, cisplatin, auranofin, metal determination, ICP-MS, lung cancer, platinum, gold

## Abstract

In this study, we developed a strategy to determine atto- and femtomolar amounts of metal ions in lysates and mineralizates of cells (human non-small-cell lung carcinoma (NSCLC, A549) and normal lung (MRC-5)) exposed to cytotoxic metallo-drugs: cisplatin and auranofin at concentrations close to the half-maximal inhibitory drug concentrations (IC_50_). The developed strategy combines data obtained using biological and chemical approaches. Cell density was determined using two independent cell staining assays using trypan blue, calcein AM/propidium iodide. Metal concentrations in lysed and mineralized cells were established employing a mass spectrometer with inductively coupled plasma (ICP-MS) and equipped with a cross-flow nebulizer working in aspiration mode. It allowed for detecting of less than 1 fg of metal per cell. To decrease the required amount of sample material (from 1.5 mL to ~100 µL) without loss of sensitivity, the sample was introduced as a narrow band into a constant stream of liquid (flow-injection analysis). It was noticed that the selectivity of cisplatin accumulation by cells depends on the incubation time. This complex is accumulated by cells at a lower efficiency than auranofin and is found primarily in the lysate representing the cytosol. In contrast, auranofin interacts with water-insoluble compounds. Despite their different mechanism of action, both metallo-drugs increased the accumulation of transition metal ions responsible for oxidative stress.

## 1. Introduction

Lung cancer is the most common type of cancer and causes many deaths (1.6 million) worldwide annually [1]. Over the past few decades, significant progress has been made in diagnostic and therapeutic approaches using new drugs or combined chemotherapy. However, lung cancer cells often exhibit radiation and chemotherapy resistance, so it is still necessary to seek new treatments [2].

Platinum-based compounds such as cisplatin (trans-dichlorodiamineplatinum(II)) are an example of significant inorganic anti-tumor complexes with good affinity to peptides and proteins that, in addition to DNA damage, can induce the formation of reactive oxygen species (ROS) and stress of endoplasmic reticulum [3,4]. Despite their frequent use, platinum-based complexes have several disadvantages, such as the development of drug resistance, many side effects, and ineffectiveness against some cancers [5]. Many newly developed metal complexes (e.g., Au, Cu, Ga, Fe, Fe, Ru) have exhibited better properties than platinum-based drugs [6]. They are mainly intended to target DNA to mimic the widely-used cisplatin.

Among gold complexes, auranofin (2,3,4,6-tetra-o-acetyl-l-thio-β-d-glycopyranosato-S-(triethyl-phosphine)-gold) deserves attention, because it shows diverse biological activity, e.g., antibacterial and antifungal properties among others [7,8]. It is due to two reactive ligands (glucopyranosate with active thiol group and triethyl-phosphine) attached to gold(I) center [9]. It is used to treat active, progressive, or destructive forms of inflammatory arthritis [10]. Recent studies investigating the cytotoxic activity of auranofin have revealed that this compound binds mainly to proteins instead of DNA [11,12]. Based on these findings, various modes of auranofin’s cytotoxicity were proposed. The gold complex was found to be responsible for the reduction of cell proliferation, oxidative stress, inhibition of synthesis of thioredoxin reductase (TrxR), deubiquitinating enzymes (DUB), and Bcr-Abl fusion protein [13,14,15].

In vitro cytotoxic lung cancer studies are usually performed using the A549 human non-small cell lung cancer cell line developed in 1972 by D.J. Giard, et al. [16]. Cells have been well characterized for years and are routinely used as in vitro and in vivo models [17]. As a reference, the MRC-5 model cell line derived from fetal lung tissue from a healthy 27-year-old woman with a genetically correct family history can be used [18]. MRC-5 lung cells, in contrast to epithelial A-549 cells, belong to fibroblasts capable of forming an extracellular matrix (ECM) [19].

The effectiveness of drugs depends on the efficiency of drug transport across the cell membrane [20,21,22]. Both drugs are responsible for oxidative stress in cells, which is usually linked to higher intracellular concentrations of transition metal ions such as iron, copper, and zinc [3,4,13,14,15]. This process is in turn strongly controlled by selenocysteine or enzymes rich in it [16,17]. It should be also mentioned that cancer cells remodel their calcium and magnesium equilibrium (important for apoptosis) to support their survival and growth [23]. Proteins of the same NRAMP family transport most divalent metal ions, so their homeostasis should reflect the degree of metabolic change in the cell [24]. Due to that, we decided to determine the amount of metal (Pt and Au specific for cisplatin and auranofin), other transition elements (Cu, Fe, Mn, Mo, Se, Zn), and redox inactive (Ca and Mg) in the model cancer and normal cells using an inductively coupled plasma mass spectrometer (ICP-MS). This highly sensitive and isotopically specific technique is commonly used to determine metals in biological tissues alongside atomic absorption spectroscopy (AAS) [25]. Although ICP-MS can detect metals below the ppt level, it requires solubilization or microwave-assisted digestion of the sample with ultra-pure chemicals and application of easy-to-clean parts in the ICP-MS system [26].

The sample purification step and enrichment of gold and platinum ions (separation techniques based on ion exchange [27]) is usually conducted to reduce the amount of Gd, Hf, and Ta in the sample (usually of geological character) as they form interfering polyatomic ions with oxygen. The efficiency of their formation can also be reduced by adding small amounts of carbon-containing compounds (i.e., methanol, glycerol, Triton-100 [28,29]), which also improves the yield of ionization and method sensitivity for gold and platinum. As the Gd, Hf, and Ta are usually not present in biological samples, other methods based on the reaction of polyatomic ions with reactive gas (O_2_ or CH_4_) or high-resolution MS are not required [26,30,31]. Unfortunately, both metal ions (Pt and Au) also cause problems due to their low solubility in aqueous solutions. This leads to adsorption of the metal ions onto various components of the sample introduction system, increased background, and reduced sensitivity. This has been prevented by adding metal ion complexing compounds such as hydrochloric acid, hydrofluoric acid, ammonium fluoride, thiourea, or cysteine to the samples or the washing solutions used after each sample analysis [28,32]. It was also noted that the replacement of the glass nebulizer and spray chamber with one made of PFA allowed for the determination of ultra-trace uranium contents in water using just a solution of nitric acid in water [33]. Teflon tubes were also proposed as suitable for an integrated sample introduction system (ISIS) [34]. Another problem is the difficult-to-define effect of sample density and viscosity on aerosol generation efficiency. In such cases, the following are used: sample dilution, the application of ultrasonic nebulizers with or without membrane desolvation, mineralization, dilution of the sample, the addition of Triton-100, centrifugation of samples, dialysis, or ultrafiltration [26,28,30,32,33,34]. The effect of other sample components on the spray efficiency can be controlled using a matrix-matching solution consisting of NaCl and CaCl_2_ or analyte-free biological material obtained from a control group [26,30,34].

Usually, the results obtained are presented as the concentration of metal in a certain volume (blood, cytosol) or the amount of metal in the dry matter of the tissue [22,35]. For in vitro cell lines tested, the amount of metal in one cell can be considered to be more appropriate. This can be obtained by LA-ICP-MS but remains problematic due to the matrix effect and sensitivity depending on the size of the evaporated sample. The second trend is the analysis of bulk biological material. Both methods should be considered complementary; one provides information on the distribution of metal ions in tissue and cells. The second enables the detection of ultra-trace amounts of metal ions that do not occur naturally in living organisms. Their toxicity often depends on their ability to enter the cell [21,22]. However, in both cases, it is necessary to control the amount of biological material. Different approaches were proposed to normalize data for metal complexes and nanoparticles: the established amount of metal in volume was recalculated against a dry mass of washed cells, the number of cells established for pellet (cells washed, trypsinized, and gently centrifuged) [36,37,38], the total amount of proteins in lysates [39] or amount of metals in the medium before/after cell culture [40]. Cell counting is implemented most frequently as it also allows changes in cell viability to be tracked.

Trypan blue is one of the most frequently used dyes to distinguish between live and dead cells in solution and establish cell density. It stains the cytoplasm of dead cells into dark blue by permeating their compromised membranes and binding to intracellular proteins. It should be noted that cell density can be inaccurately determined when viability is less than 80% due to protein aggregation [41], and a limited counting time window. It is therefore worthwhile to additionally use another method of cell staining to determine whether the determined density is under- or over-estimated.

Another problem with this type of research is the lack of certified reference materials to verify the accuracy of the metal determination method. In such a situation, it is necessary to compare metal contents obtained for the same sample by two independent analytical methods [42]. This may be difficult in the case of small quantities of biological material containing different amounts of an analyte and requiring specific storage conditions.

**Figure 1:**
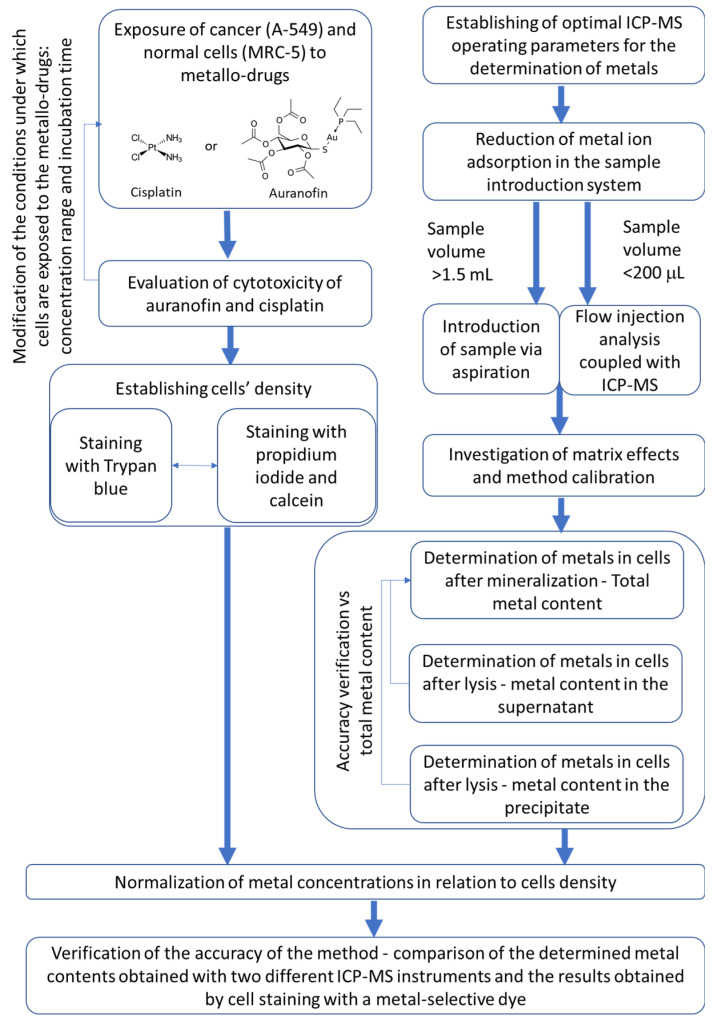
Schematic presentation of the procedure established to study the accumulation of metallo-drugs and their effects on the metal homeostasis in lung cancer and normal cells by ICP-MS.

Our study aimed to develop a method for detecting gold and platinum and other metals in cells after lysis or digestion and to normalize the data concerning cell density (established by two staining methods), allowing comparison of results obtained by two different ICP-MS instruments. We also decided to see how the method of sample introduction to ICP-MS and the degree of dilution of the biological material can affect the determinations and further compare obtained results with those obtained using typical microbiological methods. Thus, we wanted to create a strategy for the study of metal accumulation by cells based on both chemical and biological methods, emphasizing the validation of the different steps of the procedure (Figure 1).

## 2. Results and Discussion

A quantitative evaluation of cytotoxicity was carried out after a qualitative assessment of changes in cell morphology under microscopic observation. In addition, it was checked whether the final number of MRC-5 and A549 cells in the control group reached 2 × 10^4^ and 3 × 10^4^ (RSD 20%) in 1 mL after cytotoxicity tests, respectively. Otherwise, the experiments were repeated.

### 2.1. Evaluation of Cytotoxicity of Auranofin and Cisplatin

Preliminary cytotoxicity tests using MTT, based on the enzymatic activity of mitochondrial succinate dehydrogenase, were carried out for cells exposed to each of the two metal-drugs in the range 0.1–100.0 µM for 24, 48, and 72 h. The effectiveness of MTT reduction to formazan was monitored based on the change in absorbance compared to the absorbance obtained for the control group. The estimation of the IC_50_ parameter (the dose of drug responsible for 50% inhibition of the desired activity obtained for the control group) was determined according to the guidelines described by J. L. Sebaugh [43]—tested the stability of the control sample, appropriately defined curve of viability changes of cells’ (at least three points above and below bend point of the curve). Firstly, calculations were performed using Excel software. However, it was noted that the relative standard deviation for the determined IC_50_ values exceeded 40%. We have decided to increase the degree of fit of the experimental points to the curve (from r > 0.9 to r > 0.99) using Dr Fit software able to fit Hill’s equation to the points comprising the S-shaped curve (Figure S1) [44]. Thus, the relative standard deviation of the method was significantly reduced and ranged from 2 to 33% (for five independently repeated experiments).

**Figure 2:**
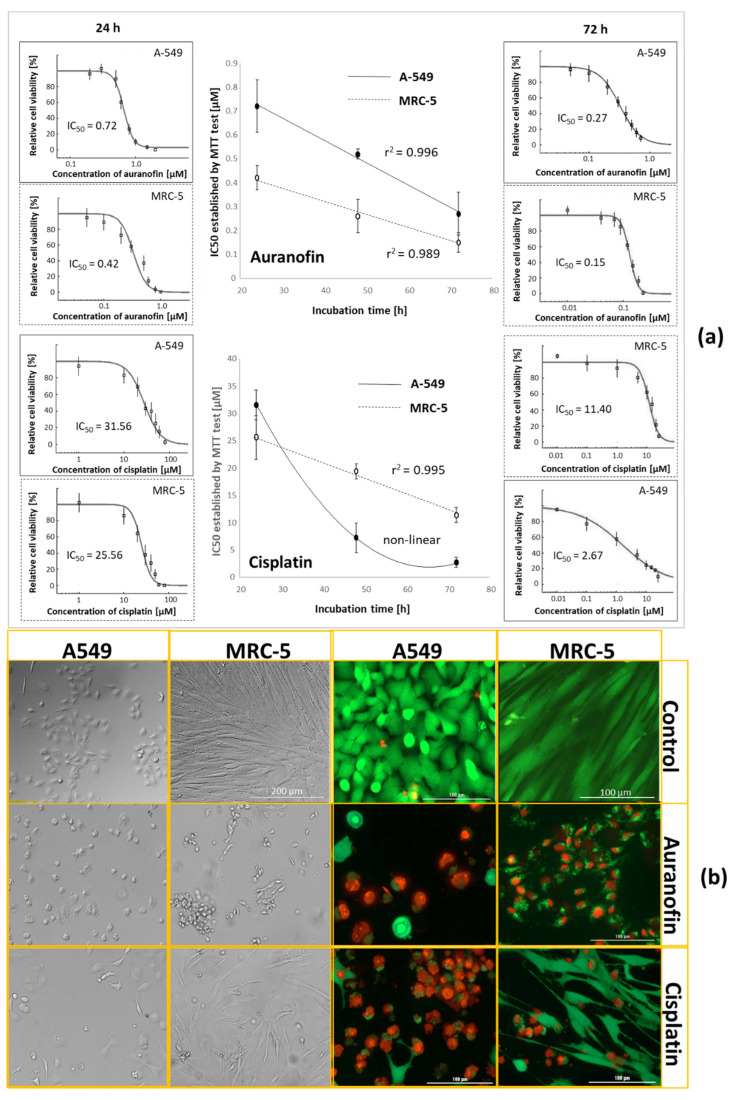
Metallodrugs (auranofin and cisplatin) concentration required for 50% inhibition of cell biochemical function (IC_50_) determined for A-549 cancer cells and normal MRC-5 cells after different incubation times and correlation coefficient (r^2^). IC_50_ was established using automatically fitted curves described by Hill equation to relative changes of absorbance obtained via MTT test (Dr Fit software) for cancer lung cells A549 and normal lung cells MRC-5 exposed to auranofin and cisplatin for 72 h (**a**). Microscopic images were obtained for lung cancer (A549) and normal (MRC-5) cells exposed to auranofin and cisplatin stained with calcein (CAM, green), propidium iodide (PI, red), and trypan blue (grey) (**b**).

It was found that the IC_50_ decreases proportionally with increasing incubation time for both tested drugs and cell lines (Figure 2, Table S1). However, it should be noted that extending the incubation time significantly increases the cytotoxicity of cisplatin to cancer cells. The intersection of the curves in Figure 2a indicates a change in the selectivity of the drug (normal vs. cancer cells), i.e., the efficiency of drug transport into the cell is most likely responsible for the change.

It should also be mentioned that the significant reduction in cancer cell viability for longer incubation times in the presence of cisplatin was accompanied by a reduction in the number of calcein-stained cells classified as viable. This means that for determining the bioaccumulation capacity of a drug by cells, it is necessary to determine the size of the cell population.

The interesting change in cytotoxicity selectivity of cisplatin from 0.8 to 4.2 (expressed as the ratio IC_50, MRC-5_/IC_50, A-549_) was not observed for auranofin. The pH of cancer cells’ extracellular matrix (ECM) microenvironment is lower than that of normal cells due to higher citric and lactic acid contents [45]. It is known that lowering the pH of the solution accelerates the hydrolysis of cisplatin [46,47] and other cytotoxic metal complexes [9,48]. Since the hydrolysis process of cisplatin is very slow compared to auranofin (minimum 48 h to reach a reaction efficiency of up to 50% [37,38], while for auranofin the reaction can be completed within few hours) and that only the formed derivatives of prodrugs are biologically active, it is possible to observe a significant change in the cytotoxicity of cisplatin (normal vs. cancer cells) given such long exposure times (24, 48 and 72 h) of cells to the drug.

Since drugs such as cisplatin can be present in the human body for up to 20 months after treatment [49], and the time required for cisplatin transformation can exceed 48 h [35,36], cells exposed to 72 h incubation were selected for further testing by ICP-MS. Based on the IC_50_ values obtained, a reduced range of concentrations of both metallo-drugs in the medium (changed from 0.1–100.0 to 0.1–25.0 and 0.1–1.4 µM for cisplatin and auranofin, respectively) was used during further studies.

### 2.2. Establishing Cells’ Density

Both auranofin and cisplatin led to a decrease in cells’ population. For this reason, cell density was monitored after each dose of the cytotoxic complex for both staining assays (Trypan blue and mixture of calcein AM (CAM) with propidium iodide (PI), Figure 2b, Tables S2 and S3). The established density of cells by both assays was in good agreement (correlation coefficient, R^2^ > 0.92). Only for normal cells exposed to auranofin was the correlation coefficient lower (R^2^ = 0.82); however, differences were not statistically significant (*p* > 0.05). Due to the good agreement of the determined cell densities and the absence of aggregates and color changes in the blanks, it was concluded that the cell density could be determined using trypan blue.

### 2.3. The Selection of Sample Introduction Method for ICP MS

**Figure 3:**
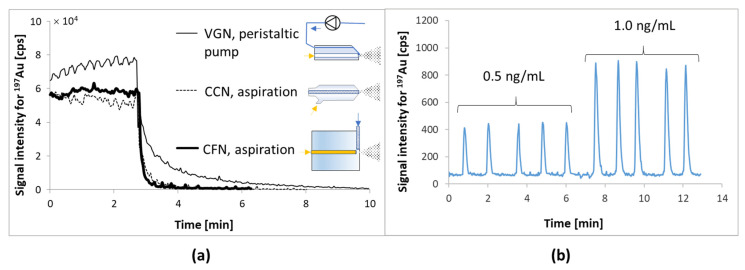
(**a**) The effectiveness of the sample introduction system rinsing with 2% hydrochloric acid solution after the introduction of gold (10 ng/mL) standard solution. VGN—V-groove nebulizer, CCN—concentric nebulizer, CFN—cross-flow nebulizer. (**b**) Changes of the signal intensity for ^197^Au during FIA injections of 0.5 and 1.0 ng/mL Au in a solution of lysate diluted in proportion 1:3 equal to 17 and 34 fg of Au per cell. A 50 µL sample injected by Rheodyne valve into 0.5 mL/min mobile phase transported via PEEK tube with ID 0.13 mm.

The determination of metals in cell lysates is a special challenge because the solution contains both gold and platinum ions as well as proteins that readily adsorb on the surface of the sample introduction elements into ICP MS. This leads to incomplete sample removal, system contamination, and an increase of the background signal from metals. Therefore, the influence of the type of nebulizer on the sensitivity of the method of determining gold and platinum was checked for diluted (1:3) cell lysates with a 2% HCl water solution (the effect of co-precipitation of metal ions with proteins was excluded because good compliance of the results for mineralized lysates was obtained—changes were in the standard deviation range). The tested nebulizers presented in Figure 3a were: V-groove (VGN), concentric (CCN), and cross-flow (CFN). In the first case, the sample solution was transported using a peristaltic pump, and the other two were used in an aspiration mode. The best detection limits (for ^195^Pt: VGN 2.3, CCN 0.1, CFN 0,05 ng/mL and for ^197^Au: VGN 1.2, CCN 0.4, CFN 0.01 ng/mL) were obtained for a cross-flow nebulizer due to the lowest noise level for baseline and the shortest washout time for gold and platinum ions from the sample introduction system in ICP MS (Figure 3a). The longest elution time for gold ions was obtained when using a VGN nebulizer, into which the sample had to be transported using a peristaltic pump. In this way, the sample had to be pumped through a tube made of Tygon, on the surface of which gold and platinum ions are adsorbed despite the use of High Purity Tygon 2275 (plasticizer-free PVC). In the case of the other two nebulizers (CCN and CFN), the sample was aspirated through a tubing made of PFA and despite extending the tubing from 30 to 70 cm, no accumulation of metal ions was observed as in the case of Tygon.

The cross-flow nebulizer operating in the aspiration mode was selected for further testing. Aspiration efficiency was controlled by monitoring the signal level of ^103^Rh, RSD <2% for *n* = 100 counts. It should be noted, however, that the use of a concentric nebulizer achieves similar detection limits and can also be used. However, this requires more caution for samples with high salt and protein content (more likely to be blocked).

Curves for Pt and Au were linear in the investigated range from 1 to 20 µg/L with R^2^ above 0.998. Limits of detection (LOD) were calculated for standard deviations (SD) of 10 measurements for blank, and it was found to be 0.05 and 0.01 µg/L, respectively (for cross-flow nebulizer working in aspiration mode). All the samples were diluted in 2% HCl water solutions and additionally in 2% HNO_3_ water solutions. Regardless of the type of acid used, the platinum and gold contents obtained in samples diluted 1:3 were not significantly different (*p* < 0.0001).

### 2.4. Sample Dilution Factor and Matrix Effect

Samples containing cell mineralizates and lysates were diluted with 2% HCl water solution in the proportions 1:1, 1:3, and 1:5. The obtained concentrations for 3 independent experiments were calculated to obtain the concentration of metals in the undiluted sample. A 1:1 dilution was found to be insufficient to reduce the matrix effect observed as signal attenuation (Table 1). On the other hand, the results obtained for 1:3, and 1:5 dilutions were consistent. Dilution of the sample also solved the problem of the minimum sample volume (≥1.5 mL) required for ICP-MS measurements infused directly and carried out with five technical replicates. A 1:3 ratio was chosen as the most suitable, offering the least variation in results. Concentrations of gold and platinum ions in solutions obtained via mineralization of cells are presented in Table 1.

The effect of sample dilution in reducing the influence of the matrix on the determination result was also checked for the other metals (Ca, Cu, Fe, Mg, Mn, Mo, Zn). As in the case of gold and platinum, the best agreement of results was obtained for a dilution ratio of 1:3 but using a solution of 2% nitric acid instead of hydrochloric acid (responsible for polyatomic interferences involving chlorine isotopes).

It was also found that samples can be diluted in a 1:3 ratio with a nitric acid solution toward the determination of gold and platinum. However, it should be noted that the measurements had to be completed within 4 h. When samples of lysates were left at 25 °C for longer periods in the nitric acid solution, the gold and platinum isotope signals decreased to about 30% of their initial heights after 24 h. Samples prepared in hydrochloric solution were stable for up to 48 h.

The developed method requires a minimum of 500 µL of purified cell lysate, which is a severe limitation (a large volume of the solution remains in the deposit after centrifugation).

### 2.5. Flow-Injection Analysis Coupled to ICP-MS

To reduce the sample volume required for the determination of metal ions, it was decided to introduce the sample as a band into a constant stream of liquid delivered (flow-injection analysis, FIA) to ICP-MS. Firstly, the HPLC system was connected to ICP-MS via a 30 cm long PEEK tube (ID 0.25 mm). The mobile phase (solution of 1% HNO_3_ in MQ-water) flow was 0.5 mL/min. The lower mobile phase flow was responsible for the peak lowering and broadening (not shown) and unstable baseline signal. The reason for this behavior is most likely due to air bubbles appearing when the CFN or CCN nebulizer aspirates the liquid at a higher velocity than is delivered by the pump. On the other hand, a higher liquid flow rate than 0.5 mL/min was also responsible for the increase in noise. A flow rate of 0.5 mL/min of the mobile phase was selected as optimal. Therefore, a PEEK tubing with an ID of 0.13 mm was installed, which narrowed the peaks and increased their height without loss of signal stability (Figure 3b).

As a result, despite a more than 10-fold reduction in the sample volume required for analysis, the detection limits for the observed metal isotopes decreased by an average of about threefold (detection limits for Pt and Au changed from 0.1 to 0.03 ng/mL). Thus, the number of cells to obtain the mineralizate or lysate was also reduced from 10^5^ to about 10^4^. Undoubtedly, such low detection limits for gold and platinum were achieved because of the rearrangement of the sample introduction system and the absence of interfering elements (i.e., Gd, Hf, and Ta) in the cells. 

### 2.6. Calibration of ICP-MS and FIA-ICP-MS and Method Validation

To investigate the effect of matrix components on signal height, the mineralizates and lysates obtained for cells from the control group were subjected to a 1:3 dilution with a nitric acid solution. Appropriate amounts of gold and platinum ions were added to such diluted solutions to obtain concentrations ranging from 0 to 50 ng/mL and a constant amount of rhodium and yttrium (10 ng/mL) solutions used as internal standards as per the Interpolative Internal Standard Method (IISM), see Section 3.9. 

The internal standard (IS) was added after mineralization and lysis and before transferring the sample to the volumetric flask. Such an approach allows minimizing errors caused by sample preparation, instrumental drift, and chemical matrix effects. In this way, changes in analyte signal intensity were tracked relative to the internal standard (I_Analyte_/I_IS_) signal, which should be constant. In practice, this is impossible, and signal variations for IS in the range of 80–120% are acceptable. 

Amounts of gold and platinum in mineralizates and lysates obtained by two methods (ICP-MS, FIA-ICP-MS) were in good agreement (within the range of their standard deviation) for both ICP-MS instruments.

The detection limits for gold and platinum were then redetermined as 0.04 and 0.12 ng/mL for lysates, respectively. After recalculating the concentrations (considering sample dilution and cell density), it was found that the method enables the detection of 3.4 and 10.2 amol of Au and Pt per cell, respectively. However, determining such small metal content (for example, 10 amol of Au per cell) is possible only if there are a minimum of 20,000 cells in 1 mL of the investigated solution (typically obtained when cells were exposed to metallo-drugs at lower concentrations). The upper limit of quantification also depends on the cell density. It may range from 33 to 500 amol/cell for Au and from 17 to 250 amol/cell for Pt when the cell density changes from 3 × 10^4^ cells/mL to 2 × 10^3^ cells/mL (Figure S2). Controlling the change in cell number (or their total mass) is necessary to validate the metal determination method for in vitro studies.

**Figure 4:**
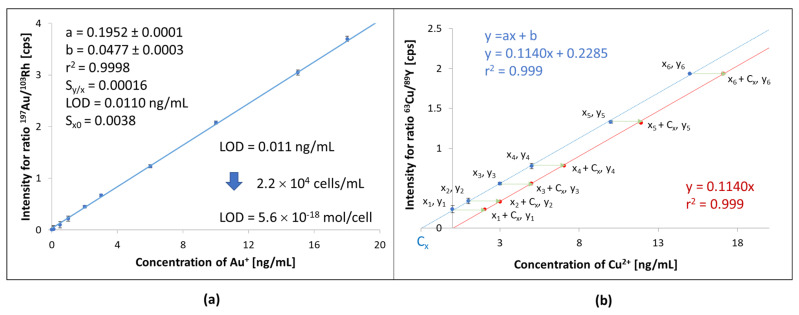
Calibration curve obtained for determination of Au, IISM (**a**) and transformation of calibration curve for determination of copper in cells’ lysate EISM→IISM (**b**). Detailed statistical characteristics of calibration curves for investigated metals is presented in Table S4.

A bigger problem was calibrating the instrument to determine other elements that naturally occur in cells. In this case, also, lysates and mineralizates obtained for cells from the control group were used, to which (after dilution in a ratio of 1:3) standard additions of metal ions were added (standard addition method, SAM) in the range of 0–50 ng/mL (C_st_ or x_i_), as was a fixed amount of yttrium or rhodium. In this way, an extrapolative internal standard method (EISM) was obtained, which was used to establish the concentration of elements in mineralizates and lysates obtained for cells from the control group (C_x_) [50,51]. After that EISM method was transferred into the IISM method by recalculation of concentrations for analytes in all solutions used for calibration (C′_st_ = C_st_ + C_x_ or C′_st_ = x_i_ + C_x_, Figure 4b). 

The calibration curve thus obtained considers the matrix’s influence on signal intensity changes (suppression or interferences). Therefore, it can be used when the investigated samples have been diluted to the same extent (dilution ratio of 1:3) as the samples obtained from cells of the control group, which were used as a solution to include matrix effects.

The established method allowed for the determination of Cu, Fe, Mn, Mo, Ni, Zn from 5 ng/mL using ICP-MS model 7500 and Ca, Cu, Fe, K, Mg, Mn, Mo, Na, Ni, Se, Zn from 0.1 ng/mL using ICP-MS with helium and hydrogen in collision cell (model HP7700) (Table S4).

Established metal amounts in lysates and mineralizates for elements determined with both ICP-MS instruments were not significantly different for cells from the control group.

### 2.7. Bioaccumulation of Auranofin and Cisplatin

Obtained amounts of Pt and Au significantly decreased the ability of cells to proliferate, especially in the case of MRC-5 cells treated with auranofin. Therefore, recalculated metal concentrations in lysates and mineralized samples considering cell density were considered as appropriate for further discussion (Table S5). This normalization of the data allowed for the comparison of the content of metals determined using HP7500 and HP7700 (He and H_2_ as collision gas), and achieved good compliance (Z-score in the range 1.2–1.9). Therefore, it was possible to compare amounts of metals obtained for different cells, and metallo-drugs in other series of experiments.

Knowing the amount of gold and platinum in the growth medium and cells, we have decided to estimate the bioaccumulation yield for metallo-drugs (DBY) using the equation:

(1) $$BY\;\left[\%\right]=\frac{Amount\;of\;metal\;in\;cells\;\left[ng\right]}{Total\;amount\;of\;metal\;in\;growth\;medium\;\left[ng\right]}\times 100\%$$

Comparing the amounts of gold and platinum in the cells against the drug content in the culture medium, the accumulation efficiency of auranofin by the cells is higher than that of cisplatin (Table 2). Undoubtedly, for this reason, the IC_50_ values were much higher for cisplatin. The accumulation efficiency decreases with the increasing concentration of both drugs in the culture medium, which is associated with decreasing number of cells.

It can be captivating that the uncertainty for established total amounts of metals and their concentrations in cell lysate increases despite higher quantities of Au and Pt in investigated samples. The uncertainty associated with the determination of metal concentrations by ICP-MS did indeed decrease. Still, at the same time, the accuracy of counting stained cells decreased for concentrations of metallo-drugs (especially for cancer cells), which was taken into account by applying the uncertainty propagation law.

Comparing the total metal contents in the cells with those determined in the cell lysates (corresponding to metal ions and water-soluble metallo-species present in intracellular space and released due to complete membrane disruption by cavitation which was checked for cells in the control group using a transmission electron microscope), more than 80% of the platinum remains in the lysate for normal cells (MRC-5), and less than 10% for cancer cells. This difference may be due to the greater ability of the MRC-5 cell to bind the platinum complex by compounds present in the cytosol (e.g., glutathione, cysteine) compared to the cancer cell. In cancer cells, tenfold higher levels of Cu, Fe, and Zn were detected, which may account for the increased oxidative stress. In addition, more efficiently hydrolyzed cisplatin in the extracellular matrix (ECM) microenvironment of a cancer cell can attach to DNA that will remain in the pellet after lysate centrifugation.

For auranofin, up to 10% of the gold taken up by the cells was detected in the lysate (Table 2). Auranofin, as it is more hydrophobic than cisplatin (logP 1.6 > logP −0.04), can be accumulated in the membrane of cells and their organelles or interact more strongly with proteins. In this case, gold compounds will remain in the pellet of the centrifuged lysate of MRC-5 and A-549 cells.

**Figure 5:**
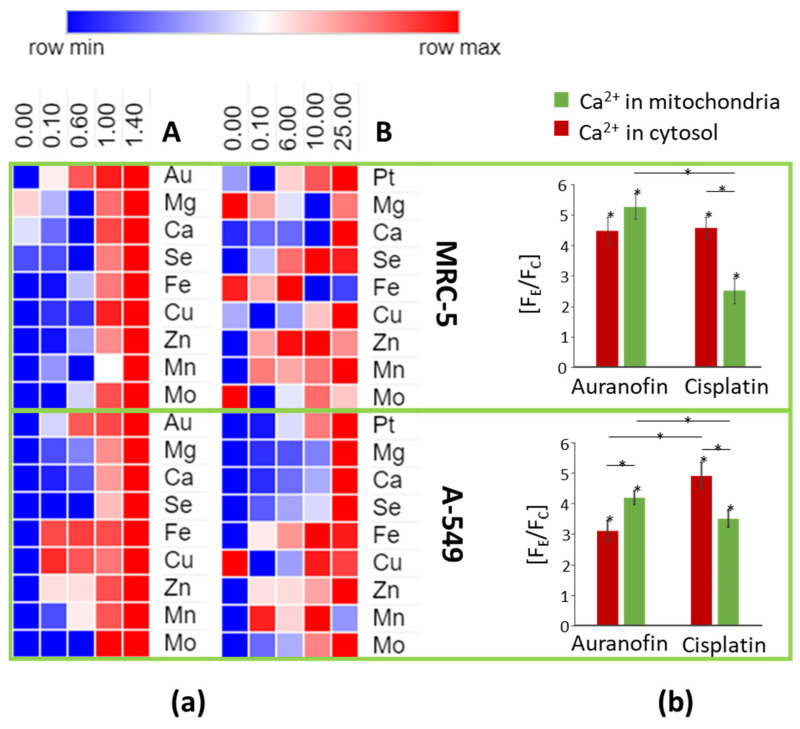
Heat-map showing changes of relative metal amounts in lysates of cells exposed to auranofin (**a**) and cisplatin (**b**) obtained with Morpheus software (https://software.broadinstitute.org/morpheus, accessed on 1 December 2021) [52]. Changes of Ca^2+^ (and other metals) amounts in cytosol and mitochondria established as the ratio of the fluorescence obtained for cells exposed to auranofin and cisplatin (F_E_) against fluorescence obtained for the control group (F_C_). *—*p* < 0.05.

It may also be noted that the amount of gold and platinum in the cells increased with the dose of metallo-drugs (Table 2 and Figure 5 and Figure S3)—represented by ascending concentrations of metals in the lysates and the mineralized cells (not shown). At the same time, an increase in the concentration of other metal ions (Ca, Cu, Fe, Mg, Se, Mn, and Zn) can be observed in the cells’ lysates and mineralizates. Only in normal cells were iron and magnesium ion concentrations decreased with the increasing concentration of cisplatin.

The accumulation of divalent metal ions in cells along with the concentration of metallo-drugs in the medium is quite surprising. We decided to verify the obtained results by applying calcium selective cell staining using Fluo-4 AM (detecting intracellular calcium levels in the 1 µM–1 mM range) and rhodamine-based dye Rhod-2 AM (detecting mitochondria calcium levels in the 1–100 µM range) [53]. It should be noted that both indicators can bind also various divalent metal cations (e.g., Cu^2+^, Mn^2+^, Zn^2+^, Pb^2+^), whereas the presence of auranofin and cisplatin did not significantly alter the fluorescence (changes in signal heights occurred within the standard deviation range of the method during preliminary tests). It was observed that the fluorescence increased from two to almost five times in the cytosol and mitochondria, confirming the results of the determinations obtained by ICP-MS, that is, the accumulation of metal ions (such as Ca, Cu, Fe, Mg, Mn, Zn) in the cell. Additionally, the smallest differences were observed for normal cells exposed to cisplatin (Figure 5). Results obtained for Ca-selective staining confirmed that cells, in addition to metallo-drugs, also accumulate increasing amounts of transition metal ions such as iron, copper, and zinc that are responsible for oxidative stress.

In addition, cellular selenium content was increased with the concentration of both metallo-drugs in the culture medium. Auranofin was responsible for more significant changes. This observation was not confirmed by any other method but is consistent with previously reported observations of the effect of gold complexes on changes in the metabolism of selenium compounds [12].

## 3. Material and Methods

### 3.1. Instruments

Fluorescent images were taken with an inverted microscope (Olympus IX71) controlled with Cell Sens Dimension software (version 1.3, Olympus). Olympus objectives (4×,10×, 20×) were used for observations. The density of cell suspensions was measured using the inverted microscope using two methods: automated—with Countess Cell Counter (Invitrogen, Carlsbad, CA, USA) and manual with Fast Read 102 counting chamber slides (Dutscher Scientific, Venice, Italy) in the case of cell aggregates created by MRC-5 cells.

Metal determination was carried out using ICP MS Model HP7500a and HP7700 (Agilent Technologies, Tokyo, Japan), and data was registered by Chemstation 10.02.B.

### 3.2. Preparation of Growth Media and Metallo-Drugs Solutions

As a growth medium Minimum Essential Medium Eagle (MEM, Sigma-Aldrich, St. Luis, MO, USA) commercial solution was used, enriched with 1 mM of penicillin, streptomycin, and 0.25 mM L-glutamine (Sigma-Aldrich, St. Luis, MO, USA). Two types of MEM enriched solutions were used: one with 10% Fetal Bovine Serum (Sigma-Aldrich, St. Luis, MO, USA) refered as FBS-MEM, and one without—referred as MEM.

Auranofin and cisplatin (Figure 1) of purity ≥98% were purchased from Sigma-Aldrich (EDQM, Strasbourg, France). A stock solution of auranofin (1.0 × 10^−4^ M) was prepared by dissolving 0.4 mg of auranofin in 100 µL DMSO and 5.9 mL of MQ-water. Next, it was diluted to obtain solutions of auranofin at concentrations: 0.1, 0.6, 1.0, and 1.4 µM in MEM. All the solutions containing auranofin were protected from light.

A stock solution of cisplatin (1.7 × 10^−3^ M) was prepared by dissolving 1.0 mg of standard in 2 mL of MQ-water. Next, it was diluted with MEM to obtain solutions of cisplatin at concentrations 0.1, 6.0, 10.0, 25.0 µM.

### 3.3. Cell Preparation

Human reference lung cancer cell line (A549, CCL-185, passage number: 1) and normal lung cell line (MRC-5, CCL-171, passage number: 30) were purchased from the American Type Culture Collection (ATCC). Cells were maintained as a monolayer culture at 37 °C in a humidified 5% CO_2_ atmosphere in FBS-MEM. In an aim to limit the influence of the number of passages on genetic drift leading even to changes of cell’s phenotype [54], cancer and normal cell lines were a sub-cultured maximum of 20 and 45 times, respectively. Cells were passaged every day (A549) or twice a week (MRC-5). To prepare proper cell suspension for the tests, cells were detached from the culture flasks using 2 mL of 0.05% trypsin-EDTA solution (trypLE Express, Gibco, Paisley, UK) for 5 min. Then cells were suspended in 6 mL of FBS-MEM and centrifuged for 5 min at 1500 rpm (Universal 32 Tabletop Centrifuge, Hettich, Tuttlingen, Germany). The supernatant was removed and cell pellets were resuspended in the proper amount of FBS-MEM to obtain the cells density of 1 × 10^5^ cells/mL.

### 3.4. Cells’ Exposure to Metallo-Drugs

Cells were transferred to the flasks with cultivating area of 25 cm^2^ and incubated for 24 h at 37 °C in a humidified atmosphere, containing 5% CO_2_ (incubator, HeraCell, ThermoFisher Scientific, Langenselbold, Germany). After incubation, the medium was removed from the flasks, and cells were washed twice with 1 mL of PBS. Different MEM solutions enriched with different amounts of auranofin or cisplatin were added to the culture flask containing 1 mln cells, each. The cultures exposed to auranofin and the control group were kept in the dark for 72 h at 37 °C in a humidified atmosphere, containing 5% carbon dioxide. The samples were prepared in three sets toward cytotoxicity tests, staining of cells, and determination of metal by ICP MS.

### 3.5. Cytotoxicity Tests

Cells were plated in 96 well plates (10^4^ cells in each) in FBS-MEM and incubated (always at 37 °C in the incubator containing 5% CO_2_) for 24 h. After acclimatization of cells growth medium was exchanged in each well plate into MEM (positive control group), MEM supplemented with auranofin or cisplatin and MEM supplemented with 0.1% Triton X-100 (negative control group). Cells were incubated for 24, 48, and 72 h. In the case of auranofin, the plates were kept in the dark including control groups. The incubation medium was removed from well plates shortly before cytotoxicity tests and cells were rinsed with phosphate-buffered saline (PBS, ThermoFisher Scientific) solution. 2.5 mg of yellow dye (3-(4,5-dimethylthiazol-2-yl)-5-(3-carboxyme-thoxy-phenyl)-2-(4-sulfophenyl)-2H-tetrazolium), MTT was dissolved in 0.5 mL PBS solution, mixed and subsequently diluted in 5 mL MEM solution, and added to each well. The plates were incubated for 4 h. Next, MEM solution was carefully removed and 200 µL of DMSO was added to dissolve formazan crystals. Absorbance at 570 nm was detected for each well. To exclude the influence of metallo-drugs on MTT test results, blank samples in wells without cells containing all components were also incubated. Obtained absorbance for blank samples was 0.108 ± 0.003 and 0.127 ± 0.002 (*n* = 6) mAU for cisplatin and auranofin, respectively. It was satisfactory low in comparison to absorbance obtained for blank samples without metallo-drugs (0.097 ± 0.004). Each absorbance was subtracted with the value obtained for blank.

To establish the viability of cells, the untreated control group of cells was considered 100% viable. The cell viability was established using the equation [55]:

(2) $$Viability=\frac{the\;absorbance\;for\;test\;group}{the\;absorbance\;for\;control\;group}\times 100\%$$

The parameter—half-maximal inhibitory concentration (IC_50_) of a drug required for 50% inhibition of cells’ biochemical function was estimated by Dr Fit software [44] using fitted dose-response curves based on Hill equation [56].

### 3.6. Cells’ Staining and Counting

The cytotoxicity of the drugs was also assessed using a commercial kit with two fluorescent dyes, calcein AM (CAM) and propidium iodide (PI) (Sigma-Aldrich). A mixture of both dyes prepared in MEM was applied according to the standard procedure established by the producer of the kit based on Johnson’s protocol [57]. Subsequently, cells were incubated for 15 min (Figure 1). Fluorescent images were taken in the spectral scanning mode at λEx/λEm = 490/525 nm for CAM and λEx/λEm = 535/617 nm for PI. Trypan blue (Sigma-Aldrich) was dissolved in MEM (0.4%), added to each vessel, and incubated for 2 min according to the W. Strober protocol [58]. Different types of cells were counted manually using Fast-Read 102^®^ hemocytometer plastic slides and automatically using hemocytometer Countess™. In addition, blank samples (without cells) were prepared for each dye and metallo-drugs to verify that they do not form complexes or aggregates that could affect the assay result.

Cells were counted in 10 different sectors of a microscopic image to obtain the average number and standard deviation for five independent series of experiments and then converted to the number of cells in 1 mL suspension.

### 3.7. Calcium Selective Cells Staining

The amount of calcium was established by using two calcium selective dyes: Fluo-4 AM—detecting intracellular calcium levels in the 1 µM–1 mM range and rhodamine-based dye Rhod-2 AM—detecting mitochondria calcium levels in the 1–100 µM range (Thermofisher, Carlsbad, CA, USA) [53]. Cells, after exposure to drugs, were transferred to 96-well plates. Next, the culture medium was collected and 100 µL of dyes mixture (1 µM each) in PBS was applied for cell staining according to the standard procedure established by the producer of the kit. Subsequently, cells were incubated for 2 h. Fluorescent images were taken using a Cytation 3 reader in the spectral scanning mode at λEx/λEm = 494/516 nm for Fluo-4 AM and λEx/λEm = 552/581 nm for Rhod-2 AM. Changes in cellular calcium levels were determined as the F_E_/F_C_ ratio, where F_E_ is the fluorescence obtained for cells exposed to metallo-drugs and F_C_ is the fluorescence obtained for cells from the control group.

### 3.8. Collection of Cells toward Lysis and Mineralization

A MEM solution, containing cells dissociated from the culture flask, was gently transferred from the flasks into 15 mL falcon tubes. The remaining adhesive cells were treated with trypLE Express (Gibco) solution for 5 min. After that cells were rinsed, suspended in 2 mL of MEM, and added to 15 mL falcon tubes. The obtained cells were centrifuged at 1500 rpm for 5 min. The supernatants were removed, and cells’ pellets were subjected to mineralization and the second set of cells was subjected to lysis.

Cells were lysed with 2 mL of deionized water using sonication using a glass-probe for 20 min with 30% of maximum power in 4 cycles. The obtained solutions were centrifuged for 20 min at 18,000 rpm at 4 °C, twice. The purified supernatant was filtered with a 0.45 µm syringe filter (Sigma–Aldrich, Bellefonte, PA, USA), two first drops were discarded, and only the remaining part of the filtrate was introduced into ICP.

For the determination of the total amount of gold and platinum, both types of cells were subjected to microwave-assisted mineralization with a mixture of 5 mL of aqua regia toward the determination of Au and Pt. For the determination of other metals, mineralization was carried out in the presence of 3.5 mL HNO_3_ and 1.5 of H_2_O_2_. The digests were diluted to a final volume of 25 mL with MQwater. Further dilutions of the digests or lysates toward ICP-MS analysis were prepared using 2% HCl solution and Rh (10 ng/mL) as an internal standard (for Au and Pt) or 2% HNO_3_ solution and Y (10 ng/mL) as an internal standard (for other metal ions).

### 3.9. Determination of Selected Metals by ICP-MS

Optimal parameters for the determination of metals were presented in Table 3. Plasma power, voltage for ion lenses, and torch position were established daily to obtain the highest sensitivity and oxide formation below 0.4% using a standard mixture of 10 ng Li, Co, Y, Ce, and Tl per 1 mL of 2% nitric acid solution. Additionally, isotopes of ^177^Hf and ^181^Ta were monitored in the samples, revealing that the contribution of HfO and TaO to the ^195^Pt and ^197^Au isotope signals used for quantification was not significant. Chloride influence, if significant, was corrected using the interference equation.

For small volumes of cells’ lysates, flow-injection analysis (FIA) was used instead of direct sample introduction. A 50 µL sample was introduced into a stream of mobile phase (5 mM ammonium nitrate) delivered by an HPLC pump (1100, Agilent Technologies, Waldbruun, Germany) to ICP-MS through Rheodyne valve and PEEK tubing.

Total amounts of metals and amounts of metals in lysates were calculated using Chemstation 10.02.B by the internal standard correction method and recalculated against one cell using cell density as the normalization factor. Statistical analysis of data was carried out using Statistica Software 13x.

## 4. Conclusions

Bioanalytical methods provide the key information necessary to design an appropriate procedure for studying the degree of accumulation of a cytostatic compound by cells: determination of the IC_50_ parameter helps to select the concentration of the compound in the medium, imaging of stained cells (in addition to showing changes in cell viability) allows for the determination of their quantity in a defined volume cell density or to confirm changes of metal amounts in cells established by ICP-MS.

The use of a cross-flow and concentric nebulizer in aspiration mode as the sample delivery method for ICP-MS, combined with sample dilution that reduces the effect of the matrix on the metals to be determined, enables the determination of gold, platinum, and other essential metals in cell lysates and mineralizates. The use of flow-injection analysis allowed similar limits of detection to be achieved, but using a tenfold smaller sample volume and thus a correspondingly smaller number of cells. FIA-ICP-MS can be used for metal determination when the amount of available sample becomes a bottleneck of the method. On the other hand, a higher number of elements to be analyzed requires a longer registration time and the ICP-MS method with nebulizer working in aspiration mode will be more appropriate. To obtain accurate assay results, it is necessary to take into account the influence of matrix components on the atomization and ionization process. This is possible by using the diluted sample as a solution modified by standard additions to calibrate the instrument. A simple mathematical transformation allows the method of standard additions to be applied to the determination of metal ions in other samples. The presence of determined metal ions in the diluted sample used as a base solution for standard solutions does not preclude this approach.

It was observed that the Pt and Au amount in the cells increased proportionally to the cisplatin and auranofin concentration in the culture medium. In addition to the accumulation of metallo-drugs, there is also an increase in the content of other metals in the cells (Ca, Cu, Fe, Mg, Zn) due most likely to the modification of the cell membrane. However, it can be noted that this process occurs differently for both metallo-drugs, ascisplatin is predominantly found in the supernatant of cell lysate consisting of water-soluble compounds (>80%) leading to more severe oxidation stress, whereas auranofin shows an affinity for water-insoluble compounds (up to 90% Au in the sediment). These changes were confirmed by increased fluorescence obtained for Ca-selectively stained cells exposed to metallo-drugs in comparison to the control group when metal-sensitive dyes for staining were used. Further studies should include the kinetics of metallo-drugs transport into the cell and its transformations in the extracellular and intercellular environment (speciation analysis).

The implemented strategy allows the application of a validated set of methods to study the accumulation of different metal-containing drugs by cells and their influence on essential metal homeostasis. Its complexity can be reduced when using certified reference materials (cytosol or freeze-dried cells with established metal content) and the isotope dilution method.

## Figures and Tables

**Table 1:** Concentrations of Pt and Au in lysates established for different dilution ratios.

Drug and Monitored Isotope	Drug Concentration in the Growth Medium [μM]	Concentration of Metal in Lysate [ng/mL] for Different Dilution Ratios (*n* = 6)		
		1:1	1:3	1:5
Cisplatin, ^195^Pt	0.1	0.23 ± 0.10	0.08 ± 0.01	0.07 ± 0.04
	25.0	12.0 ± 2.0	4.4 ± 0.1	4.2 ± 0.2
Auranofin, ^197^Au	0.1	13.1 ± 1.1	6.0 ± 0.2	6.0 ± 0.2
	1.4	115.0 ± 5.6	56.8 ± 0.7	55.2± 0.9

**Table 2:** Amounts of gold and platinum established for cells’ mineralizate and lysate (water-soluble compounds).

C_drug_ [µM]	Total Amount of Metal [Fmole/Cell] (DBY)		Amount of Metal in Cells’ Lysate [Fmole/Cell]	
	MRC-5	A-549	MRC-5	A-549
Cisplatin (Pt content)				
0.1	0.36 ± 0.11 (3%)	8.05 ± 0.70 (39%)	0.297 ± 0.059	0.036 ± 0.006
6.0	2.46 ± 0.80 (<1%)	14.87 ± 4.08 (<1%)	1.631 ± 0.264	0.913± 0.224
10.0	5.95 ± 1.29 (<1%)	35.80 ± 8.99 (<1%)	4.590 ± 0.700	1.297 ± 0.308
25.0	32.31 ± 3.10 (<1%)	49.33 ± 13.70 (<1%)	3.713 ± 0.387	2.318 ± 0.967
Auranofin (Au content)				
0.1	3.35 ± 0.57 (33%)	19.90 ± 2.26 (85%)	0.020 ± 0.005	1.660 ± 0.304
0.6	13.30 ± 1.54 (10%)	32.74 ± 5.43 (7%)	1.081 ± 0.119	1.508 ± 0.287
1.0	21.83 ± 2.61 (10%)	56.45 ± 14.95 (8%)	2.320 ± 0.237	1.609 ± 0.462
1.4	39.44 ± 5.25 (2%)	51.93 ± 12.70 (4%)	2.254 ± 0.484	2.589 ± 0.655

LOD = 0.010 fmole of Pt/cell and 0.003 fmole of Au/cell.

**Table 3:** Optimal parameters for metal determination by two ICP-MS instruments (HP7500 and HP7700).

Parameter	HP7500	HP7700
Plasma power	1310 W	1500 W
Double charged	0.1%	0.2%
Nebulizer gas flow	1.1 L/min	
Nebulizer	Cross-flow, CFN	Concentric, CCN
Sample/mobile phase flow	0.4/0.5 mL/min	
Gas (He/H2) flow	N/A	2 mL/min
Monitored isotopes	55Mn, 57Fe, 60Ni, 63Cu, 66Zn, 95Mo, 195Pt, 197Au	[He]: ^23^Na, ^24^Mg, ^39^K, ^55^Mn, ^57^Fe, ^60^Ni, ^63^Cu, ^66^Zn, ^78^Se, ^195^Pt, ^197^Au [H_2_]: ^43^Ca, ^95^Mo
Monitored ratios for isotopes for correction of interferences: [75/77 and 77/82] for Mn, Ni, and Cu; [195/177] for Pt; [197/181] for Au		
Internal standards	^89^Y, ^103^Rh	
Integration time	0.1 s	

[He]—metals determined using helium as collision gas, [H_2_]—metals determined using hydrogen as a reaction gas.

## Data Availability

Cell staining images and ICP-MS results are available from the authors on request.

## Supplementary Materials

The following are available online. Table S1. Metallodrugs (auranofin and cisplatin) concentration required for 50% inhibition of cell biochemical function (IC_50_) determined for A-549 cancer cells and normal MRC-5 cells after different incubation times and correlation coefficient (R2) for IC_50_ changes in the incubation time function. Table S2. Microscope images obtained for cells exposed to auranofin. Table S3. Microscope images obtained for cells exposed to cisplatin. Table S4. Statistical characteristics of calibration curves obtained for metal ions obtained by both ICP-MS. Table S5. Number of cells in 100 µL of cells’ suspension after exposition to metallodrugs. Figure S1. Cytotoxicity of cancer lung cells A549 (A-B) and normal lung cells MRC-5 (C-D) exposed to auranofin and cisplatin established using automatically fitted curves described by Hill equation to relative changes of absorbance obtained via MTT test (Dr Fit software). Figure S2. Number of MRC-5 (A. C) and A-549 (B. D) cells in suspension after exposition to metallodrugs. Figure. S3. Calcium selective staining of lung cancer cells (A549) and normal lung cells (MRC-5) with Rhod-5 AM dye (red) and Fluo-4 AM (green) and changes of Ca2+ amounts in cytosol and mitochondria established as the ratio of the fluorescence obtained for cells exposed to auranofin and cisplatin (FE) against fluorescence obtained for the control group (FC). *—*p* < 0.05.
