# Supplementary material for: Attomole-per Cell Atomic Mass Spectrometry Measurement of Platinum and Gold Drugs in Cultured Lung Cancer Cells
Source: Molecules. 2021 Dec 16;26(24):7627. doi: 10.3390/molecules26247627 (PMC8703441; doi:10.3390/molecules26247627)
Supplement: Supplementary file 1 [file molecules-26-07627-s001.zip › molecules-1446089-supplementary.pdf]

Supplementary electronic data

Table S1. Metallodrugs (auranofin and cisplatin) concentration required for 50% inhibition of cell biochemical function (IC_{50}) determined for A-549 cancer cells and normal MRC-5 cells after different incubation times and correlation coefficient (R^2) for IC_{50} changes in the incubation time function

**Incubation time [h]**	**A-549**	**MRC-5**
IC_{50} for auranofin [μM]		
24	0.72 ± 0.11	0.42 ± 0.05
48	0.52 ± 0.02	0.26 ± 0.07
72	0.27 ± 0.09	0.15 ± 0.04
R^2	0.998	0.994
IC_{50} for cisplatin [μM]		
24	31.6 ± 2.8	25.6 ± 4.0
48	7.2 ± 2.7	19.4 ± 1.4
72	2.7 ± 0.9	11.4 ± 0.3
R^2	0.929	0.997

Table S2. Microscope images obtained for cells exposed to auranofin

Auranofin concentration, µM	MRC-5	A-549
0, Control	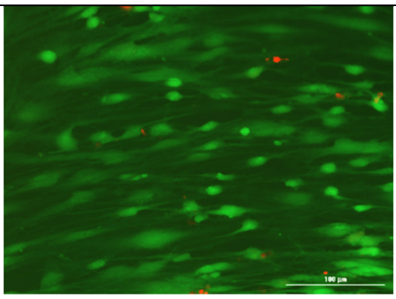	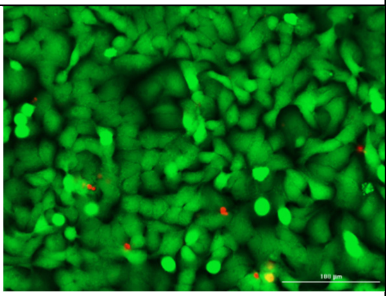
0.1	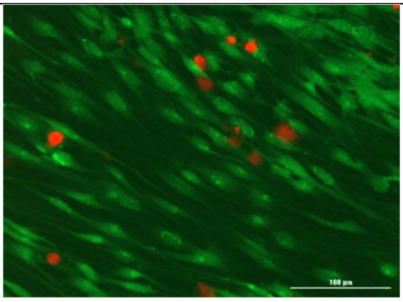	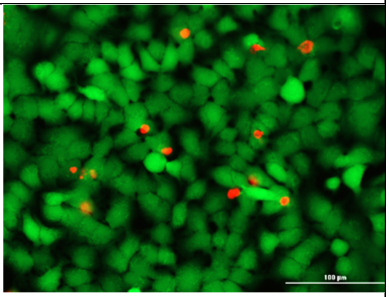
0.6	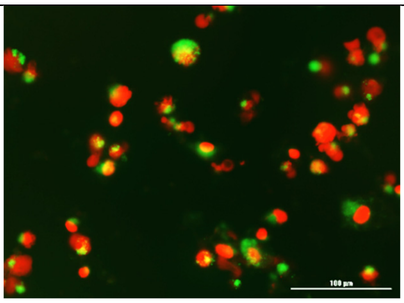	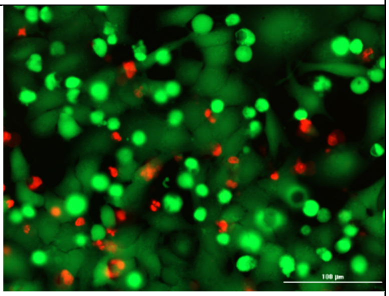
1.0	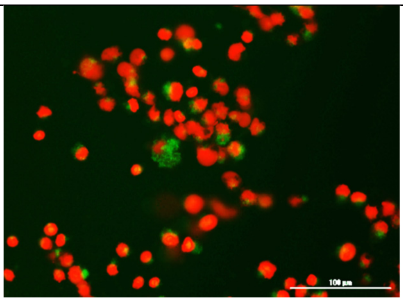	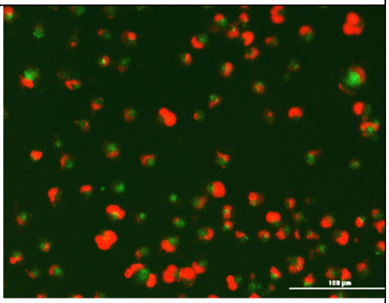
1.4	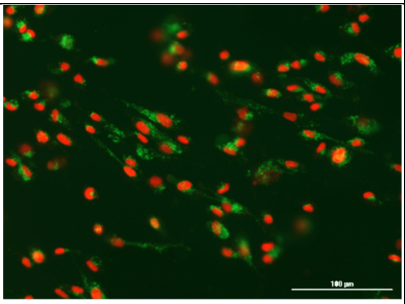	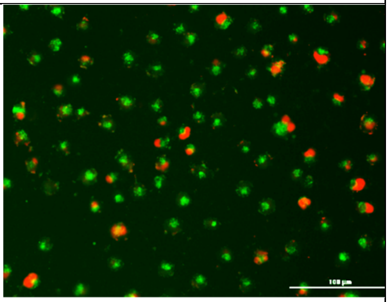
Negative control	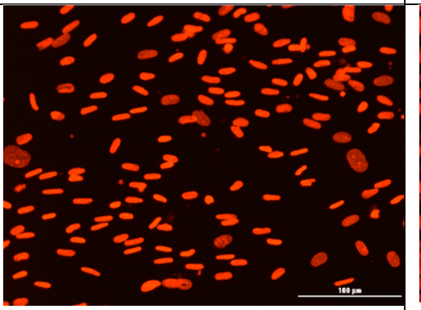	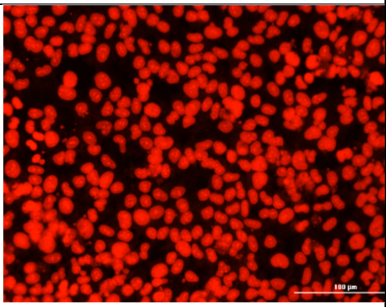

Table S3. Microscope images obtained for cells exposed to cisplatin

Cisplatin concentration, µM	MRC-5	A-549
0, Control	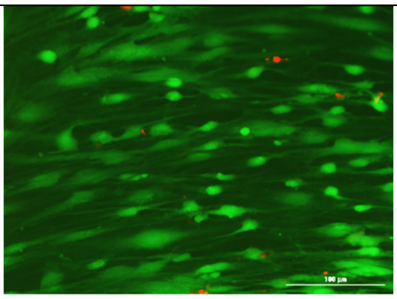	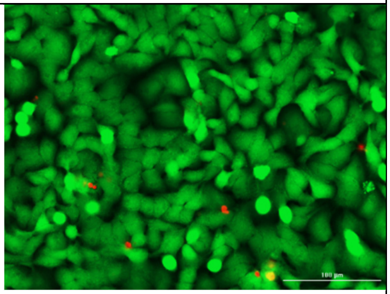
0.1	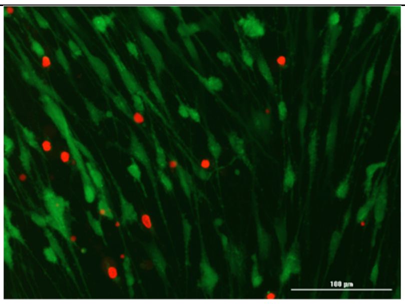	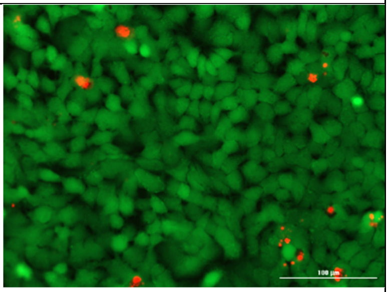
6.0	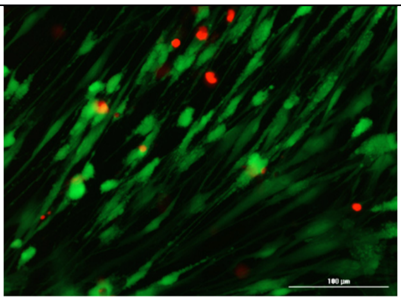	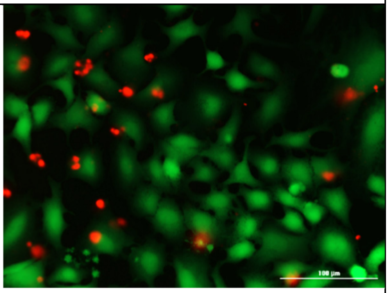
10.0	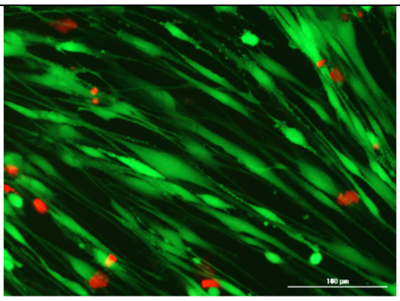	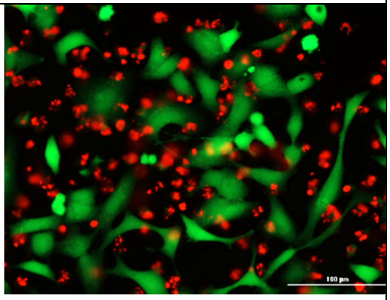
25.0	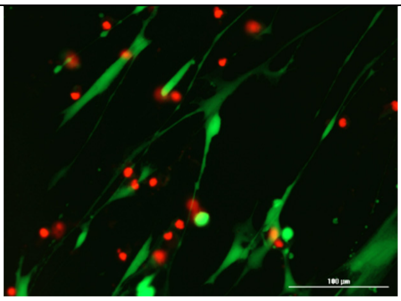	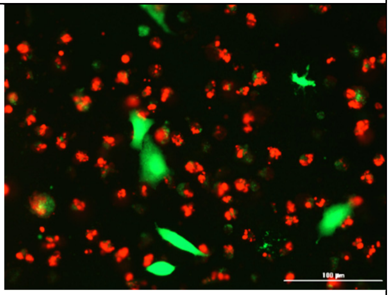
Negative control	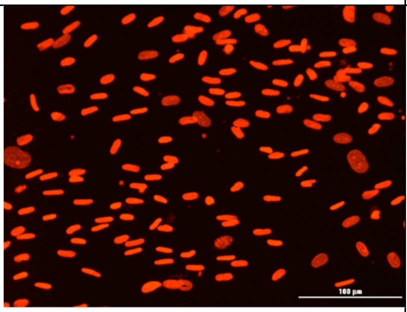	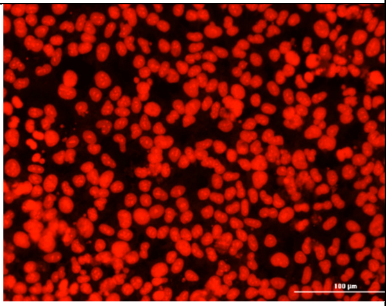

Table S4. Statistical characteristics of calibration curves (y = ax + b) obtained for metal ions obtained by both ICP-MS

ICP-MS HP7500 (cross-flow nebulizer)							
**Isotope**	**a**	**b**	**r^2**	$S_{y/x}$	**Linear range [ng/mL]**	**LOD [ng/mL]**	S_{x0}
**^{55}Mn**	0.0600 ± 0.0058	0.0646 ± 0.0062	0.997 (5)	0.0036	5 - 45	0.07	0.05
**^{57}Fe**	0.00190 ± 0.00005	0.1026 ± 0.0008	0.997 (6)	0.0006	5 - 45	0.09	0.03
**^{60}Ni**	0.0123 ± 0.0008	0.0821 ± 0.0007	0.999 (5)	0.0061	2 - 45	0.09	0.04
**^{63}Cu**	0.0281 ± 0.0009	0.0435 ± 0.0022	0.999 (6)	0.0110	2 - 45	0.04	0.02
**^{66}Zn**	0.00644 ± 0.00008	0.02970 ± 0.0021	1.000 (5)	0.0011	5 - 45	0.05	0.02
**^{95}Mo**	0.0134 ± 0.0006	0.0141 ± 0.0004	0.999 (7)	0.0051	2 - 45	0.07	0.42
**^{195}Pt**	0.0201 ± 0.0004	0.0138 ± 0.0002	0.999 (7)	0.0003	1 - 45	0.05	0.02
**^{197}Au**	0.0328 ± 0.0002	0.0084 ± 0.0001	0.998 (7)	0.0002	1 - 45	0.02	0.02

ICP-MS HP7700 with applied He and H_2 (concentric nebulizer)							
**Isotope**	**a**	**b**	**r^2**	$S_{y/x}$	**Linear range [ng/mL]**	**LOD [ng/mL]**	S_{x0}
**^{23}Na**	0.1192 ± 0.0012	2.087 ± 0.035	0.996 (5)	0.015	0 - 50	0.03	0.05
**^{24}Mg**	0.0400 ± 0.0016	0.0267 ± 0.029	1.000 (5)	0.017	0 - 50	0.13	0.41
**^{39}K**	0.0166 ± 0.0009	0.2803 ± 0.0011	0.999 (5)	0.0056	0 - 50	0.07	0.12
**^{43}Ca**	0.00016 ± 0.00002	0.00047 ± 0.00063	0.999 (5)	0.00026	0 - 50	1.05	0.59
**^{55}Mn**	0.0688 ± 0.0001	0.0026 ± 0.0029	1.000 (5)	0.0015	0 - 50	0.02	0.48
**^{57}Fe**	0.1107 ± 0.0010	0.3193 ± 0.0315	0.997 (6)	0.013	0 - 50	0.05	0.03
**^{60}Ni**	0.0413 ± 0.0003	0.0033 ± 0.0015	0.999 (6)	0.0042	0 - 50	0.04	0.83
**^{63}Cu**	0.1140 ± 0.0003	0.0094 ± 0.0033	0.999 (6)	0.0040	0 - 50	0.02	0.09
**^{66}Zn**	0.0171 ± 0.0003	0.0190 ± 0.0089	1.000 (5)	0.0033	0 - 50	0.06	0.16
**^{95}Mo**	0.0371 ± 0.0003	0.0030 ± 0.0033	1.000 (6)	0.0034	0 - 50	0.06	0.19
**^{78}Se**	0.00100 ± 0.00001	0.0001 ± 0.0006	0.995 (5)	Pierwiastek	0 - 50	1.07	0.85
**^{195}Pt**	0.1084 ± 0.0002	0.0418 ± 0.0007	0.999 (6)	0.0035	0 - 50	0.03	0.02
**^{197}Au**	0.1952 ± 0.0001	0.0477 ± 0.0065	0.998 (6)	0.0029	0 - 50	0.01	0.01

where: y – signal intensity*;* x – metal concentration in the solution, $S_{y/x}$– standard deviation for linear regression *(y-values),* S_{x0} confidence limits for concentration of analyte equal to limit of detection *(LOD);* r^2 – coefficient of determination. Confidence limits for a and b were calculated using aquation $S \times t_{(n-2)}$, where S was standard deviation for a and b, respectively

Table S5. Number of cells in 100 µl of cells' suspension after exposition to metallodrugs

Concentration of metallodrug. µM	Number of MRC-5 cells exposed to metallodrug			Number of A-549 cells exposed to metallodrug		
Auranofin	Stained with Trypan	Stained only with calcein (CAM)	Stained with propidium iodide (PI)	Stained with Trypan	Stained only with calcein (CAM)	Stained with propidium iodide (PI)
**0.0**	194 ± 9	183 ± 7	1 ± 1	291 ± 13	290 ± 8	6 ± 1
**0.1**	156 ± 10	150 ± 12	1 ± 1	188 ± 10	181 ± 9	1 ± 1
**0.6**	69 ± 10	1 ± 1	40 ± 8	125 ± 34	82 ± 23	22 ± 23
**1.0**	76 ± 8	1 ± 1	41 ± 1	66 ± 8	5 ± 5	52 ± 3
**1.4**	83 ± 7	1 ± 1	30 ± 0	31 ± 11	15 ± 10	18 ± 1
Cisplatin	Number of MRC-5 cells exposed to metallodrug			Number of A-549 cells exposed to metallodrug		
**0.0**	194 ± 9	183 ± 7	1 ± 1	291 ± 13	290 ± 8	6 ± 1
**0.1**	181 ± 5	177 ± 7	0 ± 0	185 ± 25	174 ± 15	2 ± 3
**6.0**	93 ± 18	70 ± 9	21 ± 1	130 ± 37	111 ± 17	3 ± 4
**10.0**	70 ± 10	12 ± 7	51 ± 1	130 ± 44	79 ± 51	24 ± 4
**25.0**	46 ± 6	2 ± 2	20 ± 0	63± 33	18 ± 22	31 ± 3

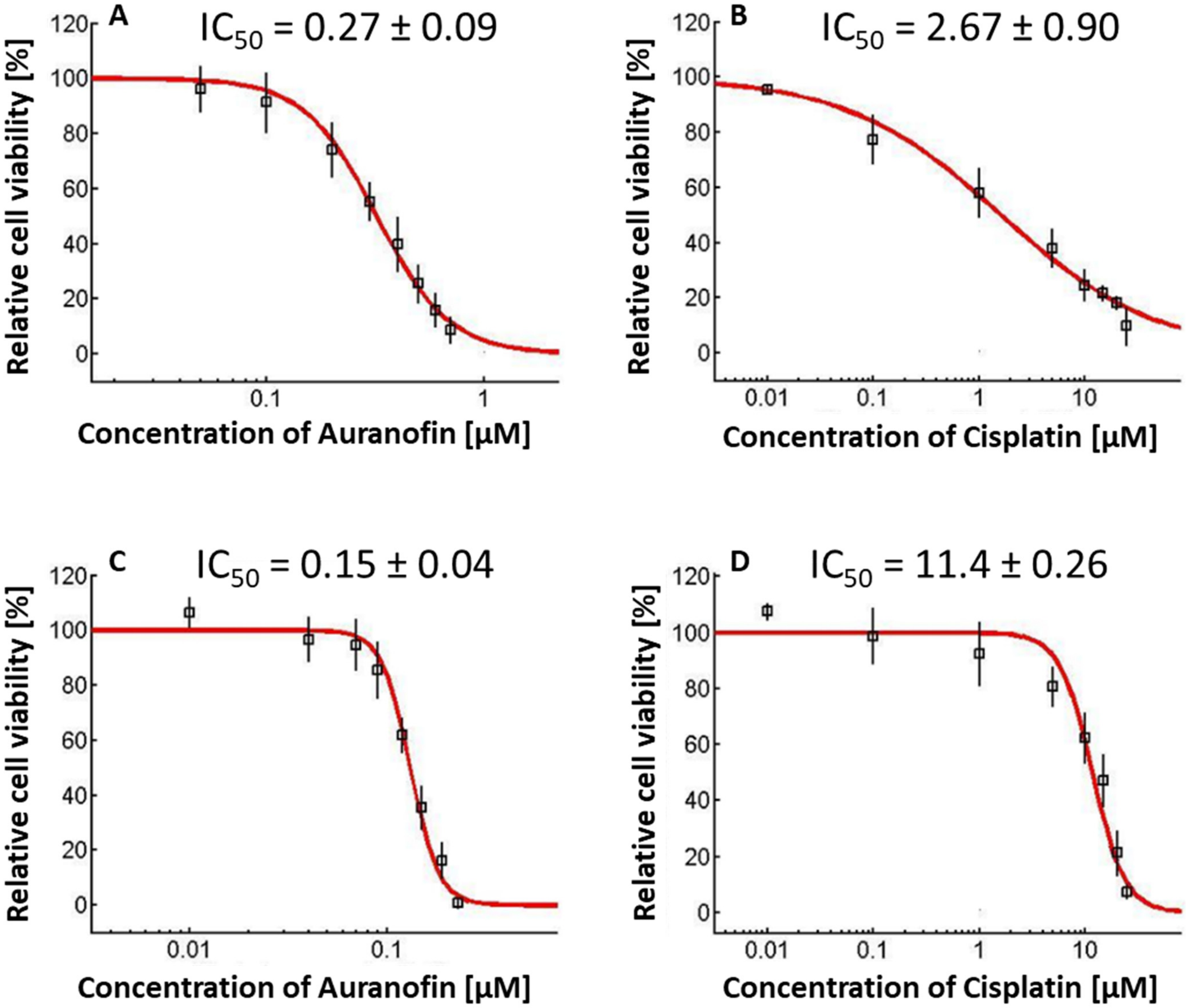

Figure S1. Cytotoxicity of cancer lung cells A549 (A-B) and normal lung cells MRC-5 (C-D) exposed to auranofin and cisplatin established using automatically fitted curves described by Hill equation to relative changes of absorbance obtained via MTT test (Dr Fit software)

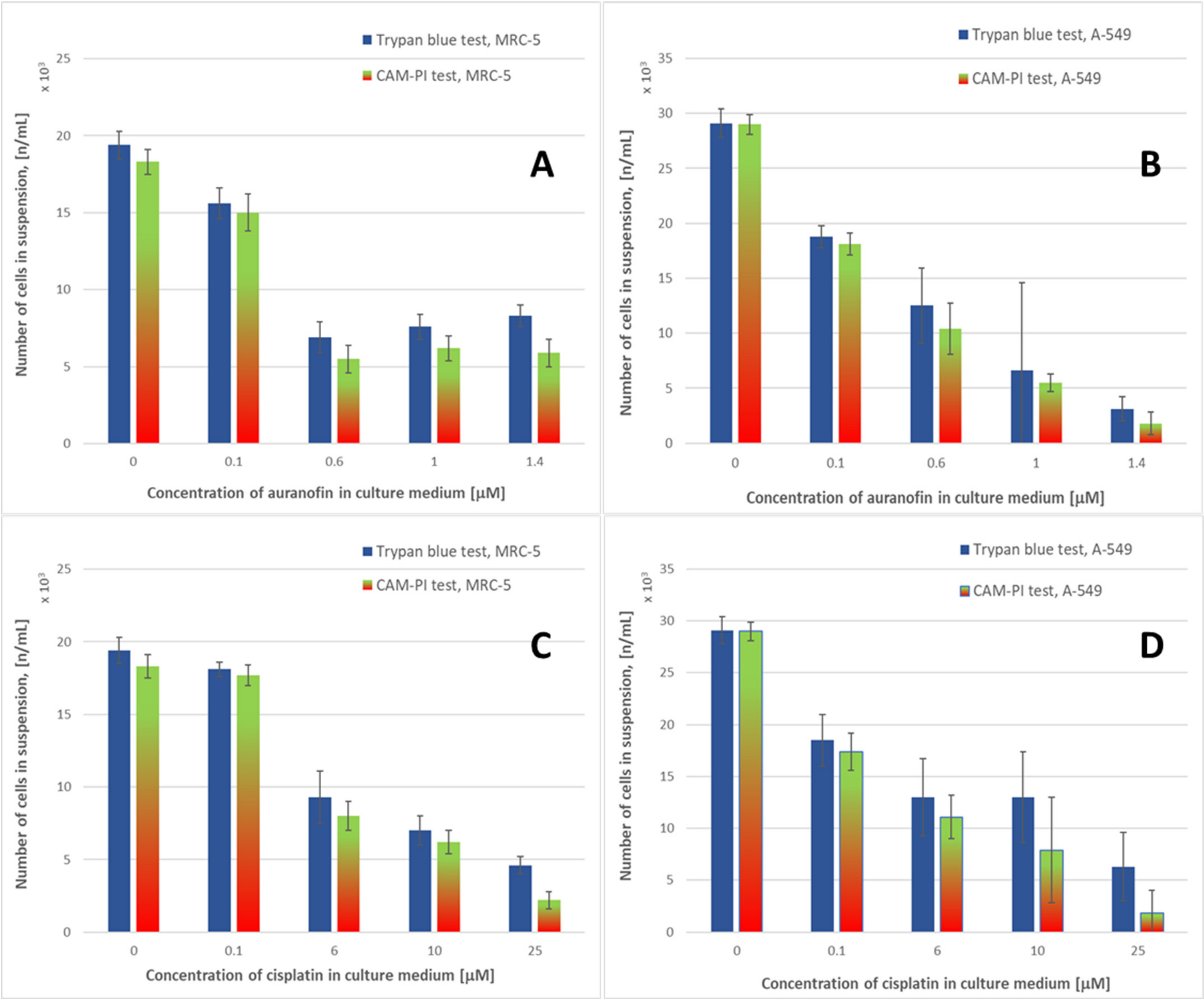

Figure S2. Number of MRC-5 (A, C) and A-549 (B, D) cells in suspension after exposition to metallodrugs

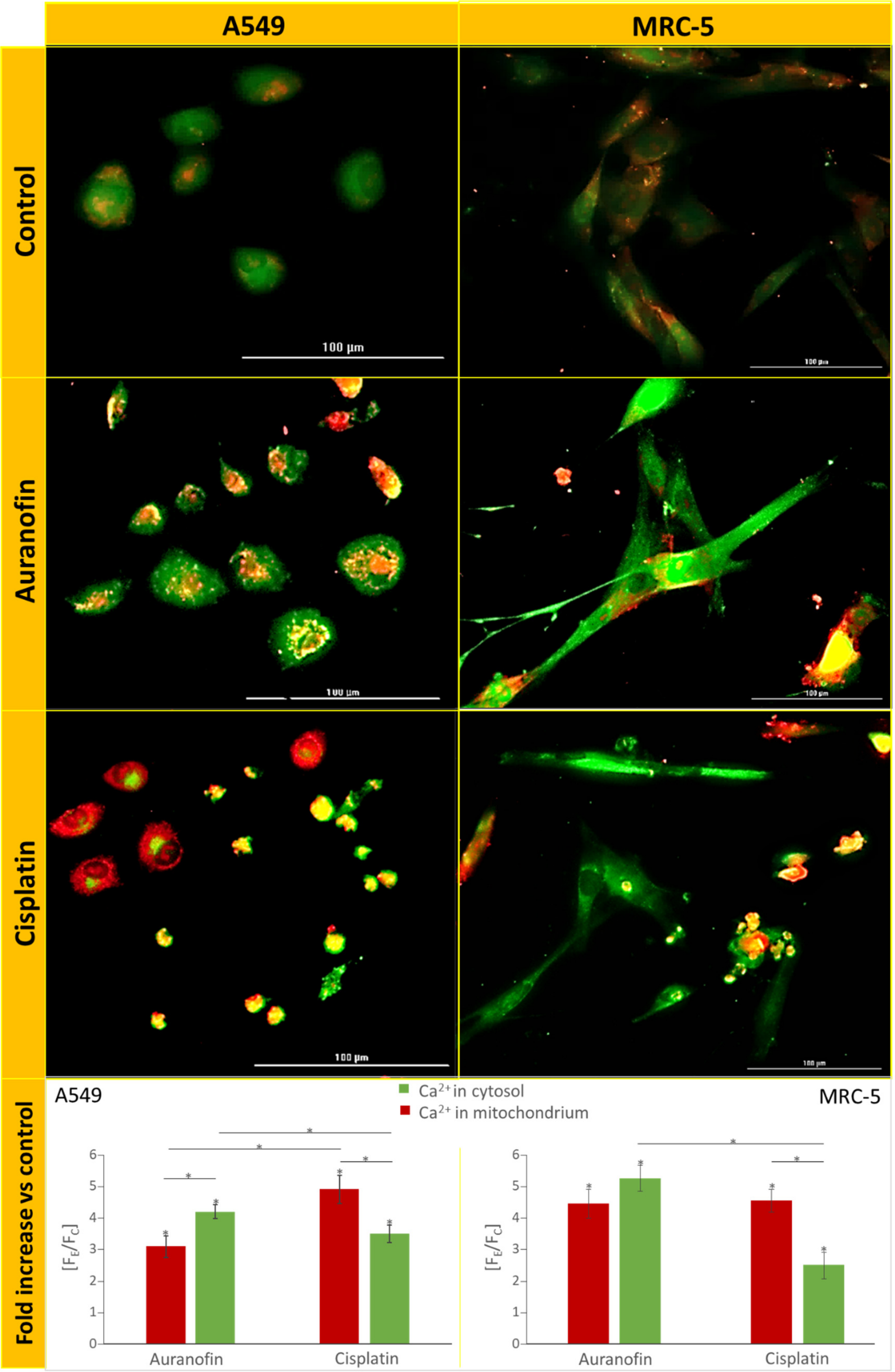

Figure. S3. Calcium selective staining of lung cancer cells (A549) and normal lung cells (MRC-5) with Rhod-5 AM dye (red) and Fluo-4 AM (green) and changes of Ca^{2+} amounts in cytosol and mitochondria established as the ratio of the fluorescence obtained for cells exposed to auranofin and cisplatin (F_E) against fluorescence obtained for control group (F_C). * - $p < 0.05$
